# Integrated Treatment to Achieve Functional Recovery for First-Episode Psychosis

**DOI:** 10.1155/2012/962371

**Published:** 2012-05-10

**Authors:** Marcelo Valencia, Francisco Juarez, Hector Ortega

**Affiliations:** ^1^Division of Epidemiological and Psychosocial Research, National Institute of Psychiatry “Ramón de la Fuente”, Calzada Mexico-Xochimilco 101, Colonia San Lorenzo Huipulco, 14370 Mexico City, Mexico; ^2^Division of Clinical Services, National Institute of Psychiatry “Ramón de la Fuente”, Calzada Mexico-Xochimilco 101, Colonia San Lorenzo Huipulco, 14370 Mexico City, Mexico

## Abstract

This study describes an integrated treatment approach that was implemented to enhance functional recovery in first-episode psychotic patients. Patients were randomized to two treatment conditions: either to an integrated treatment approach: pharmacotherapy, psychosocial treatment, and psychoeducation (experimental group: *N* = 39) or to medication alone (control group: *N* = 34). Patients were evaluated at baseline and after one year of treatment. Functional recovery was assessed according to symptomatic and functional remission. At the end of treatment, experimental patients showed a 94.9% of symptomatic remission compared to 58.8% of the control group. Functional remission was 56.4% for the experimental group and 3.6% for the control group, while 56.4% of the experimental group met both symptomatic and functional remission criteria and were considered recovered compared to 2.9% of the control group.

## 1. Introduction

Schizophrenia-spectrum disorders are characterized by the presence of psychotic symptoms, cognitive deficits, poor quality of life, and psychosocial deterioration [1–4]. Of all illnesses that afflict humans, schizophrenia is considered the 7th most disabling [5]. While perhaps only 1% of the population has schizophrenia, 30% experience an onset of psychotic symptoms by age 18 [6, 7]. Most patients are likely to experience multiple episodes of acute symptomatology, causing severe long-term functional impairment [8]. Relapse can be expected in 70% of patients after the first episode [9].

Intervention strategies for first-episode psychosis include implementation of pharmacological treatment and managing side effects. Psychoeducation is relevant for optimizing the management of side effects and promoting compliance with medication. Once the acute episode has been resolved, emphasis should focus on relapse prevention. The next stage would include implementation of psychosocial intervention facilitating compliance with medication, learning the warning signs of relapse, managing stressors, solving family problems, plus preparing to reestablish social relations and work or school activities that were interrupted [10, 11]. Pharmacological and psychosocial treatment strategies offer hope of preventing progression to long-term psychosis and moving toward a new recovery model for early psychosis. It is aimed at improving psychosocial functioning and promoting independent living in the community.

Two key elements have been considered in the recovery model: symptomatic remission and functional recovery. Antipsychotic medication normally is prescribed for first-episode psychotic patients. They usually are young patients, and a great majority of them are responsive to pharmacotherapy [12]. Research shows that remission of psychotic symptoms generally occurs in 50% of individuals within the first three months [13], 33% [14] and 75% within six months [15], and 83% after one year of treatment [16]. A complete picture of symptomatic remission and functional recovery can be consulted in [12, 17]. In addition to pharmacological treatment, psychosocial interventions have been designed for first-episode psychosis. Young patients experiencing psychosis may benefit from various approaches: cognitive therapy, social skills training, supportive therapy, housing, vocational assistance, family therapy, assertive community, and family Psychoeducation [12]. These services are delivered for in- and out-patients, individually in a group format, family therapy, and case management.

Specific goals are set, such as improving social functioning [18], quality of life and cognitive functioning [19], prevention of relapse [20], compliance with medication [21], and reducing trauma secondary to psychosis and hospitalization [22]. Evidence shows the efficacy of adjunctive psychosocial interventions for first-episode psychosis [23–30]. Methodological complications have been found since most published studies utilized quasi-experimental and single-group designs to assess the effectiveness of treatment programs [12]. In a meta-analytic review only three randomized controlled trials were found that met rigid research criteria [31]. Randomized controlled trials for first-episode psychosis could be worthwhile.

A recently introduced definition for remission in schizophrenia by the “Remission in Schizophrenia Working Group” (RSWG) in the USA [32] by Andreasen and colleagues has generated considerable interest and opened a new perspective for assessing remission with specific criteria. Symptomatic remission was defined according to a threshold of severity with a score of mild or less using eight key schizophrenia symptoms that represent the “core features” of the illness on the Positive and Negative Syndrome Scale (PANSS) [33]. A period of six months must be maintained as a minimum time threshold to achieve remission. New remission criteria have also been introduced in Europe [34–37] which include the terms “response and remission” that can be assessed either with the Brief Psychiatric Rating Scale (BPRS) [38], or the Positive and Negative Syndrome Scale (PANSS) [33]. Although new criteria for remission in the USA and Europe have been well accepted by clinicians and researchers, no consensus has yet been reached for an internationally accepted definition of remission. Remission rates vary between 17% and 88% [39] according to a recent review of 13 studies that include first-episode psychosis and use the Andreasen criteria [32].

On the other hand, recovery has gained relevance since the introduction of specific criteria to assess functional recovery for schizophrenia. According to Liberman et al. [40, 41], the definition of recovery should include symptom remission, full- or part-time activities in work or school, independent living without day-to-day supervision, seeing friends on a regular basis, and being financially independent for at least two consecutive years. Torgalsbøen [42] Torgalsbøen and Rund [43] consider that the definition of recovery ought to include a reliable diagnosis of schizophrenia at an earlier time, criteria for diagnosis not fulfilled at present, being out of hospital for at least five years, not being on antipsychotic medication or only on low dosage, and showing psychosocial functioning within a “normal range” >65 on the Global Assessment Scale. International consensus still has not been reached about the concept of recovery, which complicates the assessment of this variable. However, recovery includes symptomatic and functional remission, implying a return to “normal psychosocial functioning” [44, 45] that can be measured with the following mean scores: >50 [46], > de 61 [47], and >65 [42] according to the Global assessment of Functioning Scale [48].

Achieving recovery in schizophrenia includes three components: (1) “response”, indicated by maintaining stability; (2) “remission”, as improvements in cognition, functioning, and quality of life; (3) “recovery” as being functional and demonstrating social autonomy [49]. The efficacy of psychosocial interventions combined with pharmacotherapy has created new expectations about the possibility of first-episode psychotic patients achieving functional recovery. Recovery rates for first-episode psychotic patients have been reported at 48% [46], 50% [50], 19.2% [51], and 31% [17]. In another study, however, 79.8% of first-episode patients did not show functional recovery [15].

This paper reports findings after a one-year randomized controlled trial of an early-psychosis-integrated program consisting of pharmacological and psychosocial treatment for patients and Psychoeducation for relatives, compared with a standard care of pharmacotherapy alone. Patient outcome at the end of treatment was compared in terms of symptoms, psychosocial functioning, relapse, rehospitalization, compliance with medication, and therapeutic adherence. In addition, two components, symptomatic and functional remission, were assessed as indicators of functional recovery [49]. Therefore, we chose as an operational definition of functional recovery the combination of symptomatic remission according to the Andreasen criteria (eight specified PANSS items requiring a score of ≤3: mild or less: for at least 6 months) [32], and functional remission according to the Torgalsbøen criteria with a GAF score above 65 [42, 43]. The one-year time period for patients in the treatment program was considered to be the duration criterion to achieve functional recovery.

Goals of the interventions included (1) training patients to acquire social skills, (2) improving psychosocial functioning, (3) preventing relapse and rehospitalization, (4) promoting treatment compliance, and (5) achieving functional recovery measured by symptomatic and functional remission.

## 2. Methods

### 2.1. Participants and Study Design

**Figure 1:**
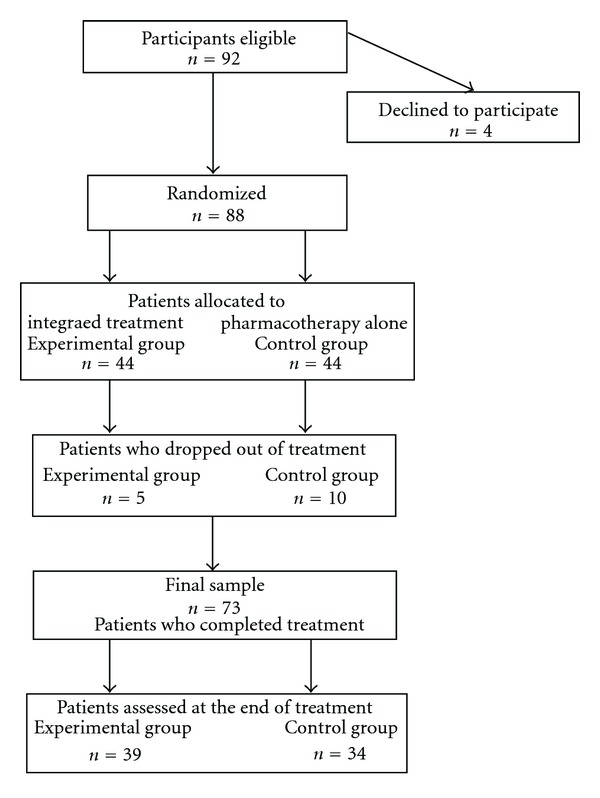
Participants flow chart.

 Participants included first-episode patients who had never been treated before. These outpatients were receiving pharmacological treatment at the Schizophrenia Clinic of the hospital of the National Institute of Psychiatry in Mexico City. Of the 92 patients who met eligibility criteria, four refused to participate (all four accepted exclusively pharmacological treatment), and 88 consented to participate in the study. They were randomly assigned to two treatment conditions: 44 patients to the integrated treatment program (experimental group) and 44 to medication alone (control group). Of the 88 patients who began treatment, five from the experimental group (11.3%; two started full-time jobs, two returned to school, and one moved out of Mexico City for family reasons) and ten patients from the control group (22.7%; five moved out of Mexico City for family reasons, three decided to receive treatment in another psychiatric hospital, and two for unknown reasons: it was not possible to locate them by phone, telegram, or in person at the community houses they left) failed to complete the study protocol with a total of 15 patients (17%) for the total sample. The final sample was 73 patients: *n* = 39 in the experimental group and *n* = 34 in the control group. The participant flow chart is shown in Figure 1. Patients were evaluated at baseline and after 12 months of treatment.

First-episode psychotic patients were recruited into the study when they met inclusion criteria and were taking anti-psychotic medication for the first time, allowing a period of no more than 15 days to demonstrate that they were clinically stable to participate in the treatment program. Their diagnoses were verified according to the DSM-IV [52] criteria and corroborated with the CIDI [53]. Study protocol participants had to meet these inclusion criteria: be receiving antipsychotic medication, be clinically stable in terms of psychotic symptoms (corroborated by a score lower than 60 in the PANSS), have completed at least six years of elementary education, be between 16 to 50 years old, have no substance (drug or alcohol) abuse verified with their relatives before and during treatment, and be living with their families in Mexico City's metropolitan area.

### 2.2. Procedures

Before any procedure was performed, the study protocol was approved by the Research Committee and the Ethics Committee of the National Institute of Psychiatry. Patients and relatives were informed about the treatment program. After they agreed to participate, they voluntarily expressed in a written informed consent document their desire to participate in the research project. Patients were then administered instruments, described in Section 2.4. Measures assess symptomatology, symptom remission, psychosocial functioning, and functional recovery at baseline and at the end of treatment. Separate raters completed the ratings. Later, patients and their relatives were randomly assigned to the integrated treatment program or to continue with pharmacological treatment alone.

### 2.3. Interventions

 The integrated approach was composed of the following interventions.

#### 2.3.1. Psychosocial Treatment

 The design process of the treatment program included the identification of clinical and psychosocial problems of patients, as well as family members' needs and demands. An exploratory study was conducted that included 42 participants: 16 clinically stabilized patients, 16 of their relatives, and 10 mental health professionals (two psychiatrists, two clinical psychologists, two psychiatric nurses, two psychiatric social workers, and two schizophrenia family therapists). Focus groups were conducted with patients, relatives, and mental health professionals (6–10 participants per group). A consensus was reached about clinical and psychosocial problems for first-episode psychotic patients. Exploratory study results identified several clinical and psychosocial problems of patients: does not know his/her diagnosis (90–95%), does not know the characteristics of the illness (95–100%), does not need medication (90–95%), does not need psychotherapy (95–100%), unemployed (45–65%), lack of financial resources (50–75%), economically dependent upon his/her family (70–80%), does not have friends (40–50%), does not have a loving relationship (70–80%), and does not have good family relations (60–70%). All patients were receiving antipsychotic medication. In addition, various areas were identified where patients had difficulties that interfered with their community-functioning medication and symptom management, social and family problems. Therefore, learning certain skills was set as a goal, that is, medication compliance, acquiring knowledge about the illness, identifying warning signs of relapse, developing a relapse preventive plan, developing skills to manage social relations, and learning problem-solving skills for better family relations. Various therapeutic modalities were recommended as components of an integrated and comprehensive mental health system including antipsychotic medication, psychosocial treatment, psychoeducation, and family therapy.

Psychosocial treatment included these four areas: (1) medication management, (2) symptom management, (3) social relations, and (4) family relations. All are described in a therapist's manual that includes the skills corresponding to each area, plus training strategies for each session [54]. Two therapists taught patients skill acquisition using the “learning activities” [55–57]. The seven proposed learning activities were reduced to six, since video technology used in the United States has not yet been developed in Mexico. Learning activities included (1) introduction and explanation of skills to be learned in each session, (2) skill demonstration by therapists that included a question-and-answer segment for clarification of skills to be learned, (3) patient practice of skills using role playing and other techniques, (4) feedback allowing patients to identify resources needed to use skills in the real world, (5) practice skills in the community, and, (6) each session began with verification of skills registered in a learning checklist. A therapist evaluation form was used to verify that all treatment areas were conducted properly. Therapists' competency during treatment was assessed by a specially trained research assistant. Before treatment, competency levels had to be demonstrated with at least a 90 percent level of efficacy. Monitoring for maintenance of fidelity occurred throughout the study. Group sessions, six patients per group, were conducted weekly by two therapists with a time limit of 75 minutes during one year of treatment.

#### 2.3.2. Psychoeducation

 This intervention was mandatory for at least one relative per family who received information during ten multifamily group sessions about the illness, symptoms, medication management, side effects, compliance, keeping appointments, and recognition and management of warning signs of relapse. In addition, four sessions for each patient and his family were held oriented to problem solving and improving communication skills. Two family therapists were in charge of Psychoeducation and family sessions.

#### 2.3.3. Pharmacological Treatment

Patients of both groups received medication management at the Schizophrenia Clinic of the National Institute of Psychiatry. Two clinical psychiatrists, who were blind to the two treatment conditions, gave patients 20-minute monthly consultations, registered attendance, controlled prescription of antipsychotic medication, and verified compliance with medication during one year of treatment.

Professional participants in the treatment team included two psychiatrists for medication management, two clinical psychologists in charge of psychosocial treatment, and two family therapists for Psychoeducation and family sessions.

### 2.4. Measures

Symptomatology was assessed using the Positive and Negative Syndrome Scale (PANSS). This is a validated 30-item scale, Spanish adaptation [58], consisting of three subscales: positive (7 items), negative (7 items), and general psychopathology (GPS; 16 items). Each item is scored from 1 (absence of psychopathology) to 7 (extremely severe).

Symptom remission was assessed according to the Andreasen criteria [32] for symptomatic remission using eight items of the Positive and Negative Syndrome Scale (PANSS): P1 (delusions), P2 (conceptual disorganization), P3 (hallucinatory behaviour), N1 (blunted affect), N4 (social withdrawal), N6 (lack of spontaneity), G5 (mannerisms/posturing), and G9 (unusual thought content). All scores have to be 3 (mild) or less during six months.

Psychosocial functioning was assessed using the Global Assessment of Functioning Scale (GAF) [48]. This instrument measures symptom severity and the degree of impairment in psychological, social, and occupational functioning on a mental health-illness continuum which indicates the level of functioning ranging from 1 to 100. Scores above 65 are considered within the functionally recovered range [42, 43].

Separate raters were in charge of evaluation of both groups under study at baseline and at the end of treatment. Raters received necessary training for proper application of all research instruments; they were blind to which study group a patient belonged to and were instructed to remind patients to abstain from mentioning what type of treatment they were receiving. Raters did not participate in the treatment team and had no knowledge of the research project.

During treatment, relapse, and rehospitalization rates, compliance with medication and therapeutic adherence were evaluated for all participants. When patients experienced warning signs of relapse with significant exacerbation of psychotic symptoms, they received immediate consultation from their treating psychiatrist, who then made necessary adjustments in their antipsychotic medication to avoid relapse. A psychotic relapse was registered when patients had at least a 20% worsening on the PANSS total score from baseline evaluation. Similar relapse criteria have been used by other researchers [59, 60]. When psychotic symptom exacerbation could not be controlled or stabilized with antipsychotic medication, the patient was admitted to the hospitalization unit. This was registered as a rehospitalization. Medication compliance was assessed by the treating psychiatrist during monthly consultations for pharmacological control with the patient and a relative participating in psycho-education. Compliance was assessed if the patient took at least 90 percent of prescribed medication; otherwise, nonadherence was assessed.

### 2.5. Statistical Analysis

 Data analysis included the following: descriptive and Chi square analysis to compare percentages, Student's *t*-tests to verify that there were no significant differences between the two groups under study in their initial levels of symptomatology, and psychosocial functioning. At baseline, Student's *t*-tests were utilized to verify that no statistically significant differences existed between the two groups regarding the PANSS and GAF scores. Analysis of variance (ANOVA) for repeated measures detected before-after differences within and between the two study groups. Standardized estimate of effect sizes were calculated using Cohen's *d* formula [61] defined as $d={\overset{®}{x}}_{1}-{\overset{®}{x}}_{2}/s$, where ${\overset{®}{x}}_{1}$ and ${\overset{®}{x}}_{2}$ are the means at baseline and at end of treatment of the two groups under study and *s* is the pooled intra group standard deviation (SD). For assessment of effect size, three levels were considered: small = 0.25, medium = 0.50, and large = 1.00 irrespective of the sign (+ or −) of the number [62]. Data analysis was carried out using the Statistical Package for Social Sciences (SPSS) for Windows 11.5 [63].

## 3. Results

Baseline characteristics of the sample are shown in Table 1. Patients in both treatment conditions were similar with no demographic differences for any of these variables, except for occupational status with a small percentage of unemployed patients in the control group. No statistically significant differences were found at baseline between the two groups under study in symptomatology, (PANSS) or psychosocial functioning (GAF). There were no differences between the two groups under study in the use of typical versus atypical antipsychotic medication (*X* ^2^ *P* > 0.05) or in medication dosage (*t* *P* > 0.05). Significantly statistical improvements in symptomatology were found over 12 months of treatment according to mean changes scores, as rated by the PANSS, in positive and negative symptoms, general psychopathology, and in total PANSS score for both groups under study. Group-by-time analysis demonstrated significantly greater improvement in patients of the experimental group when compared with patients receiving medication alone. Comparison of the effect sizes was large for the experimental group on the total PANSS score, positive scale, negative scale, and in the general psychopathology scale. Effect sizes were medium for all score scales of the control group. Significant improvement in psychosocial functioning was also found for patients of the experimental group but not for patients of the control group since they remained at the same level of functioning (41–50) as rated by the GAF, from baseline to posttreatment assessment. Patients of the experimental group improved two levels of functioning from 41 to 50 at baseline to 61–70 at the end of treatment. Effect size was large for the experimental group and small for the control group (Table 2).

In addition, relapse and rehospitalization rates as well as medication compliance and therapeutic adherence were measured during treatment. At the end of treatment lower relapse (10.3%; *P* < .01) and rehospitalization rates (5.1%) were found in the experimental group compared to 35.7% and 10.7%, respectively, for the group that received medication alone. Compliance with antipsychotic medication was higher in the experimental group (85% versus 67.6%) of the control group (*P* < .01). Therapeutic adherence to the psychosocial treatment sessions was 87.2%, which means a higher adherence level according to the therapeutic adherence levels: excellent: 90–100; high: 80–89, good: 70–79, regular: 60–69, poor: 50–59, and bad: 40–49. 

The assessment of symptomatic and functional remission as well as functional recovery demonstrated at baseline that symptomatic remission was achieved by 33.3% of the experimental group compared with 20.6% of the control group. At the end of treatment experimental patients showed a 94.9% of symptomatic remission compared to 58.8% of the control group. Functional remission was achieved by 56.4% of patients of the experimental group compared to 3.6% of the control group at the end of treatment, while 56.4% of the experimental group met both criteria: symptomatic and functional remission at the end of treatment and were considered recovered compared to 2.9% of the control group (Table 3).

## 4. Discussion

Conclusions from the present study show that patients who received the integrated approach demonstrated statistically significant improvements in symptomatology, psychosocial functioning, lower relapse and rehospitalization rates, higher compliance with medication, and high therapeutic adherence. Improvements can be seen, for example, considering one variable such as medication compliance related with relapse prevention. In this study, medication compliance for the integrated treatment group was high (85%), demonstrating that these patients learned the necessary skills about symptom and medication management that helped prevent relapse: their relapse rate was low (10.3%). 

These results indicate that outcome can be improved through early intervention after the onset of psychosis. Moreover, it has been established that maintenance of a stable clinical state is no longer considered the ultimate goal of treatment. The new focus of treatment includes achievement of symptomatic and functional remission, establishing functional recovery as the final goal of treatment. In the present study, implementation of an integrated approach (pharmacotherapy, psychosocial intervention, and psychoeducation) demonstrated that half of the patients recovered, proving that this modality of treatment can be helpful in enhancing functional recovery in first-episode psychotic patients. We conclude that, in the return to clinical stability, symptomatic and functional remission might lead to improving functional recovery. Models for integrated care have demonstrated their effectiveness through research updates and systematic reviews of the literature in numerous countries in various outcome variables such as improving psychosocial functioning, cognitive deficits and quality of life, acquisition of social skills, preventing relapse, and optimizing satisfaction with treatment [64–67]. It also has been considered that integrated approaches “show promise for improving functional recovery for schizophrenia patients” [67]. 

In the last ten years a research line relating the integration of treatment and rehabilitation approaches for chronic schizophrenia patients has been conducted at the National Institute of Psychiatry, in Mexico City. We carried out several experimental and clinical trials comparing various treatment groups during six months or twelve months of treatment [68–70] and more recently with first-episode psychotic patients as reported in the present study. To achieve our goals, patients and relatives were considered as healthy allies and collaborators of the treatment team, reaching a consensus between patients, relatives, and the research team about patient's clinical and psychosocial problems and relative's needs and expectations. Relatives' participation was considered as a key element since approximately 95% of our patients live with family members [71]. The emphasis was on establishing a therapeutic alliance with patients and their relatives. Two major concerns in treating patients with early psychosis are relapse and noncompliance. Poor medication compliance can lead to relapse and rehospitalization. It has been recommended that sustained psychosocial interventions over at least one year of treatment are needed to maintain the goal of relapse prevention [10]. Although relapse might be considered as an indicative of a deteriorating course of the illness, we offered quick intervention and proper resolution that was seen as an excellent opportunity for patients and relatives to learn about relapse and to acquire necessary strategies to prevent relapse, especially if patients are young and relatives inexperienced with mental health services. A study showed that, when patients were asked about their needs, learning how to prevent relapse was considered a priority [72]. 

Early psychosocial interventions for first-episode psychosis could be used as preventive measures to avoid further complications such as chronicity and disability that have been found in the long-term course of schizophrenia. Evidence-based practices indicate that there is a consensus regarding the components of intervention that should be considered for first-episode psychosis which include a combination of effective pharmacological and psychosocial interventions. Issues that need to be sorted out in the future include finding an international consensus about symptomatic remission and addressing considerations such as how recovery is defined and how recovery is assessed. Because this consensus was lacking, an operational definition was developed for the present study to assess functional recovery. How long patients should remain in early treatment programs is a question that needs to be addressed. In our case, a limitation of the present study has been that we have not been able to carry out, due to financial reasons, the corresponding followup to participants of the treatment program. The continuing of care would include followup as a necessary step for monitoring clinical and psychosocial functioning of patients in the community. In the last fifty years there has been a substantial change and a reevaluation in the treatment of schizophrenia. In addition to antipsychotic medication, psychosocial interventions have been recognized as an important component of a therapeutic approach to achieve recovery in early psychosis.

## Figures and Tables

**Table 1:** Sociodemographic and clinical characteristics of the participants.

	Experimental *n* = 39	Control *n* = 34
Gender, *n (%) *		
Male	30 (76.9)	25 (73.5)
Female	9 (23.1)	9 (26.5)
Marital status, *n (%) *		
Single	39 (100)	34 (100)
Occupation, *n (%) *		
Employed	6 (15.4)	8 (23.5)
Housewife	1 (2.6)	1 (2.9)
Student	2 (5.1)	7 (20.6)
Unemployed	30 (76.9)	18 (52.9)
Age, years, $\overset{®}{X}(s)$	24.5 (3.0)	24.1 (3.2)
Education, years, $\overset{®}{X}(s)$	10.7 (1.9)	10.5 (2.0)
Age at onset, years, $\overset{®}{X}(s)$	18.7 (3.2)	19.5 (3.5)

**Table 2:** Symptomatology (PANSS) and global functioning (GAF) of the study groups at baseline and at the end of treatment.

	Experimental *n* = 39	Control *n* = 34	Statistics^b^		
			Main effect for time	Main effect for group	Interaction of group and time
PANSS^a^ overall score, $\overset{®}{X}(s)$					
Baseline	86.9 (41.1)	78.8 (35.6)	*P* < .001	—	*P* < .01
Post treatment	40.2 (9.6)	52.7 (15.7)			
Effect size	−1.1	−.7			
PANSS positive^a^, $\overset{®}{X}(s)$					
Baseline	19.2 (10.9)	17.0 (10.2)	*P* < .001	—	—
Post treatment	8.5 (2.0)	10.4 (4.0)			
Effect size	−1.0	−.6			
PANSS negative^a^, $\overset{®}{X}$(*s*)					
Baseline	23.1 (11.5)	21.5 (10.1)	*P* < .001	—	*P* < .01
Post treatment	10.4 (3.9)	14.6 (6.3)			
Effect size	−1.1	−.7			
PANSS GPS^a, c^, $\overset{®}{X}$(*s*)					
Baseline	44.6 (20.6)	40.2 (17.0)	*P* < .001	—	*P* < .05
Post treatment	21.3 (4.6)	27.6 (8.1)			
Effect size	−1.1	−.7			
Level of global functionig^d^ (GAF), $\overset{®}{X}$(*s*)					
Baseline	44.2 (6.8)	44.7 (6.9)	*P* < .001	*P* < .001	*P* < .001
Post treatment	68.0 (9.3)	46.6 (10.2)			
Effect size	3.5	.28			

Notes: ^a^higher scores indicate more severe symptoms, ^b^analysis of variance for repeated measures,

^c^GPS: general psychopathology scale, ^d^higher scores indicate better global functioning.

**Table 3:** Symptomatic remission, functional remission, and functional recovery at baseline and at the end of treatment.

	Experimental *n* = 39	Control *n* = 34	McNemar
Symptomatic remission at baseline^a^	13 (33.3)	7 (20.6)	*P* < .01
Symptomatic remission after treatment^a^	37 (94.9)	20 (58.8)	

Functional remission at baseline^b^	—	—	
Functional remission after treatment^b^	22 (56.4)	1 (3.6)	*P* < .01

Functional recovery at baseline^c^	—	—	
Functional recovery after treatment^c^	22 (56.4)	1 (2.9)	*P* < .01

^a^Andreasen criteria.

^b^Torgalsboen criteria.

^c^a + b.
